# Development of abnormal diagnosis and factory evaluation standards for RV reducer based on vibration characteristics

**DOI:** 10.1038/s41598-025-90302-x

**Published:** 2025-02-17

**Authors:** Xin Wang, Chunlei Li, Suben Lin

**Affiliations:** 1 School of Intelligent Manufacturing and Electronic Engineering, Wenzhou University of Technology, Wenzhou, 325025 China; 2 School of Mechanical Engineering, Baoji University of Arts and Sciences, Baoji, 721016 China; 3 Hengfengtai Precision Machinery Co., Ltd, Wenzhou, 325024 China

**Keywords:** RV reducer, Vibration characteristics, Fault diagnosis, Factory inspection standards, Engineering, Mechanical engineering

## Abstract

Vibration characteristics are important reference indicators for reflecting the overall performance of RV reducers. At present, there is no factory testing standard for vibration characteristics applicable to production enterprises in the industry. This study conducted vibration characteristic tests on RV40E reducers at different speed ratios. Through a large number of sample comparisons, suitable vibration characteristic factory testing standards for production enterprises were provided. Detailed on-site inspection standards have been developed based on research and analysis results for defective products such as eccentricity, gear defects, and detection errors. This standard can cover 90% of defect types within the factory. The samples are specific and executable. This study solves the problem of the lack of clear vibration detection standards in the industry. For the first time, such a detailed sample set of internal data within a company has been provided. It has extremely practical engineering application value and provides data reference for the development of RV reducer industry.

## Introduction

RV reducer is a precision transmission component in the industrial field. The machining accuracy requirements for its various components are very high. Partial dimensions are required to meet the fourth level machining accuracy standard. Minor errors in each component can cause differences in performance^1^. Therefore, high consistency in processing and assembly is required. Although studies have shown that the impact of various machining errors on overall performance varies in magnitude^2^. However, various processing and assembly errors cannot be completely eliminated. Even leading companies in the industry still cannot accurately control the quality consistency of their products. There is also a lack of unified universal testing standards in the industry to evaluate whether products are qualified or not. Each enterprise can only establish its own testing standards to determine whether the product is qualified or not. Indicators such as transmission error, stiffness, backlash, and efficiency^3^ can be directly tested and evaluated through a test bench. Noise is usually detected using a noise meter. However, periodic or occasional abnormal noises such as tooth bumping cannot be detected by a noise meter. It still needs to rely on manual judgment. This creates a lot of uncertainty for factory evaluation.

In order to eliminate the uncertainty of human judgment and provide a unified testing standard, this study used vibration characteristic analysis and combine a large amount of product test samples to propose a qualified judgment standard for RV reducer products based on vibration monitoring. The fault category of non-conforming products was identified to facilitate subsequent repairs.

Some scholars have conducted research on the vibration characteristics of RV reducers. Vibration analysis were conducted from the aspects of experiment^4^, nonlinear dynamic characteristics^5^, and random vibration^6^. Theoretical modelings were also essential, such as traditional torsional dynamics models based on time-varying stiffness^7^, as well as simulations of tooth profiles and errors^8^. Of course, it is also necessary to verify the theoretical model through dynamic simulation^9^ and finite element analysis^10^. Finally, a method similar to that proposed in reference^11^ was formed to determine different operating conditions. So far, although a large amount of theoretical research has been conducted from the perspective of vibration, these achievements have not been practically applied in engineering. If these theoretical studies can be transformed into experimental testing methods with practical engineering application value, it will fundamentally guide and improve the product performance of enterprise production.

In order to propose specific and achievable factory application testing standards, this article conducted real-time product vibration performance tracking for the RV reducer production line. Several high proportion abnormal states have been summarized, and specific evaluation criteria applicable to factory assessment have been provided. This standard has been implemented in the factory. Ultimately, the testing plan will be integrated into the automated production line to achieve automated testing of factory products.

This article has focused on studying the vibration characteristics of products and determining the standard judgment of vibration characteristics. In the future, machine learning^12^, EEMD-MPA-KELM^13^, neural networks^14^ and other methods will be used to apply the research results of this paper to achieve automatic determination and classification of fault types. Ultimately, it can form automatic product testing.

To ensure consistency in product performance, the author conducted assembly consistency research on the production line before conducting the experiment. The selective assembly of various components of the RV reducer was carried out using genetic algorithm^15^, which improved the consistency of product assembly. At the same time, efforts were made to ensure that the processing and manufacturing quality of the components was qualified^16^. Errors caused by processing and assembly issues should be eliminated as much as possible. In the process of judgment, this article only provides judgment indicators for a few types of abnormal states that account for a large proportion during factory inspection. There are also some types of faults that are caused by fatigue after running for a period of time, such as root cracks^17^, bearing damage^18–20^, wear loss^21^, stability^22^ and other fault characteristics, which are not within the scope of this study. The author will gradually add and improve in subsequent research. This article provided preliminary vibration testing standards applicable to production, covering 90% of the operating conditions found on site. For more types of low probability operating conditions, we will supplement and improve them in subsequent experimental runs.

Based on a large number of basic experimental tests, this article summarizes the general testing standards that are currently lacking in the industry. The results of this article will help improve the performance of RV reducers and guide process improvements. This research has been applied to small-batch testing on the production line, achieving manual detection. Subsequently, the testing standards will be perfected and large-batch testing on the production line will be realized. Moreover, a system software based on these theoretical indicators will be developed to achieve automated monitoring and automatic diagnosis. All samples in this article come from actual processing errors on the production line, not from laboratory simulations. The basic data test results shared in this article provide a data reference for the industry and have strong practical value.

## Experimental testing equipment

All testing works for this article were completed in the company’s factory inspection laboratory, and all samples were sourced from products produced on the production line. All component errors were actual errors in the processing process, with no errors intentionally created. This article focused on testing the most widely used product on the market, the RV40E. In conjunction with the factory’s existing factory inspection indicators such as noise and dimensions, the vibration test results were compared to summarize the product performance inspection standards. The experimental test bench and reducer parameters used are as follows.

### Test bench

**Fig. 1:**
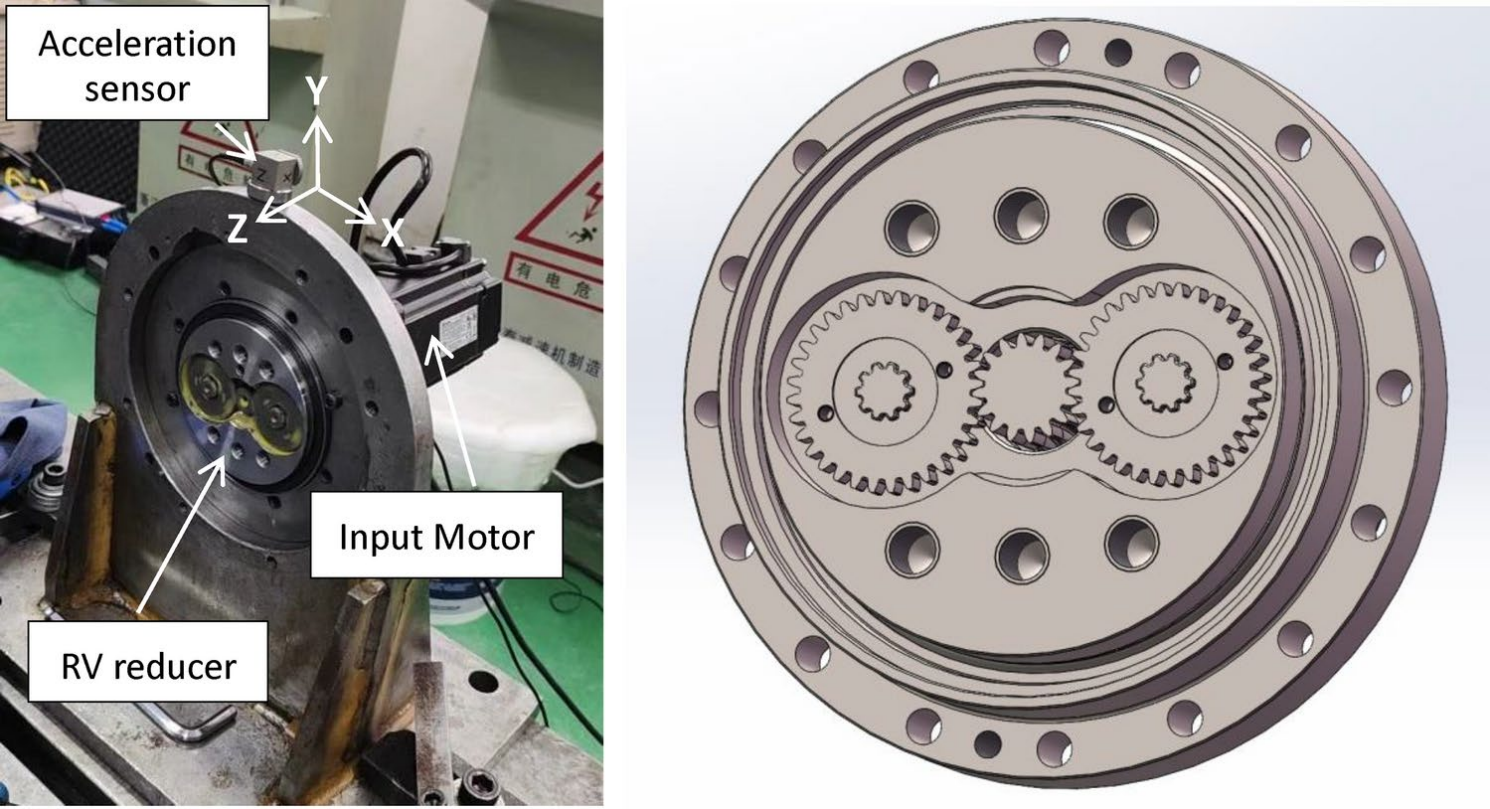
RV reducer factory test bench.

This article focused on the testing and research of RV40E series reducers. The test bench is shown in Fig. 1, with the RV reducer fixed on the bracket, and the motor connected to the RV reducer for direct testing. After measuring and comparing multiple positions, the accelerometer was finally placed above the fixed bracket for vibration testing. The test signals in three directions at this position were relatively clear, with X being the horizontal direction, Y being the vertical direction, and Z being the axial direction. The sensitivity of the three-way acceleration sensor is 100 mV/g, the measurement range is 1–10 kHz, and the sampling frequency is 5000 Hz. The installation method was magnetic attraction.

### RV reducer parameters

**Fig. 2:**
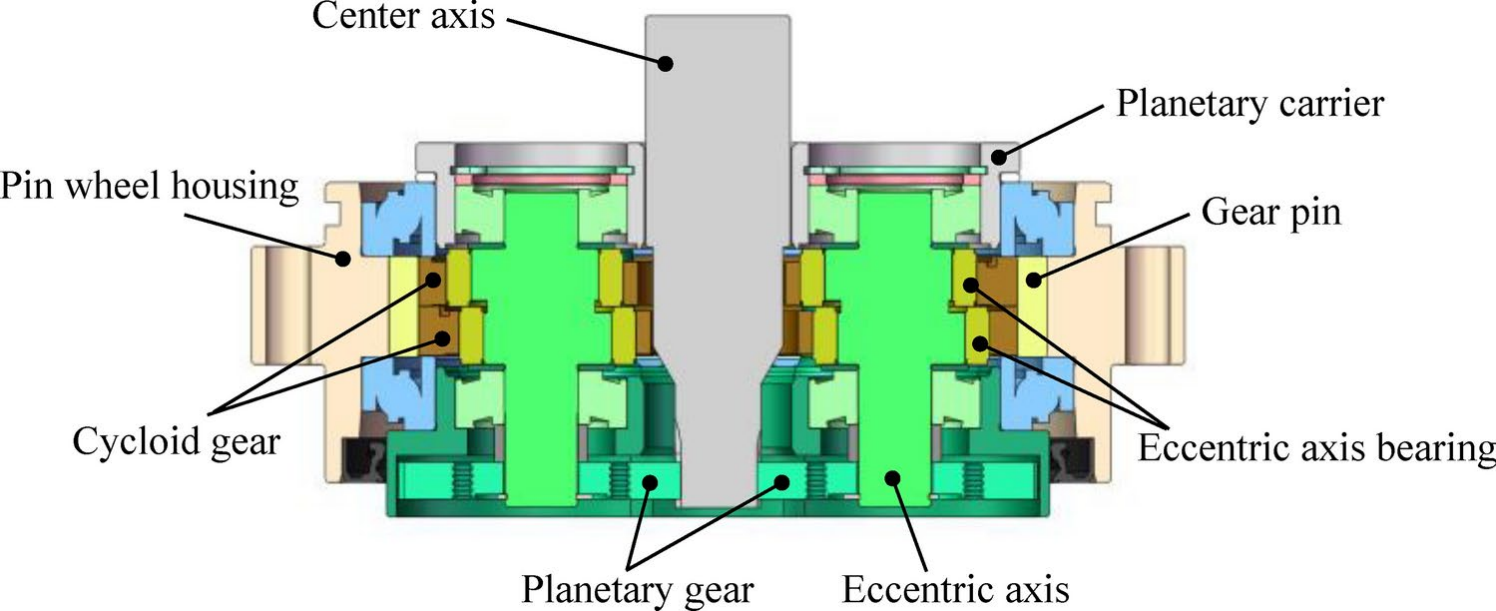
RV reducer transmission diagram.

The structure of the reducer tested in this article is shown in Fig. 2. The meshing between the central axis and the planetary gears is the first stage meshing, while the meshing between the gear pin and the cycloid gear is the second stage meshing. The motor is connected to the center axis as the input end. The RV40E reducers are mainly sold in four speed ratios in the market. The numbers of teeth on each gear at different speed ratios are shown in Table 1. Due to the use of actual samples from the factory for testing, samples of all four speed ratios may occur in different types of faults. The code we usually use to describe samples is product specification—speed ratio, where RV40E-121 represents the model RV40E with a speed ratio of 121.

**Table 1:** Number of gear teeth for each speed ratio of RV40E.

Number of teeth	Speed ratio *r*			
	81	105	121	153
Central axis *z*_1_	16	10	12	15
Planetary gear *z*_2_	32	26	36	57
Cycloid gear *z*_3_	39	39	39	39
Pin wheel housing *z*_4_	40	40	40	40

All tests in this article were conducted using an input speed *v*_1_ of 2000 rpm and repeated in both forward and reverse directions. During testing, the main focus is on issues such as vibration, noise, and abnormal sounds. Table 2 shows the characteristic frequencies at different speed ratios when the input speed was 2000 rpm. These characteristic frequencies were used in the time–frequency analysis in Chapter 3.

**Table 2:** Characteristic frequencies of gears at all stages (Hz).

Characteristic frequency	Formula	*r* = 81	*r* = 105	*r* = 121	*r* = 153
Central axis rotation frequency *f*_1_	$f_{1} = \frac{{v_{1} }}{60}$	33.33	33.33	33.33	33.33
Planetary gear rotation frequency *f*_2_	$f_{2} = \frac{{z_{1} z_{4} }}{{\left( {z_{3} - z_{4} } \right)\left( {z_{1} + z_{2} z_{4} } \right)}}f_{1}$	− 16.46	− 12.7	− 11.02	− 8.71
Cycloid gear rotation frequency *f*_3_	$f_{3} = \frac{{{\text{z}}_{1} }}{{{\text{z}}_{1} + {\text{z}}_{2} {\text{z}}_{4} }}f_{1}$	0.41	0.32	0.28	0.22
Second stage meshing frequency *f*_2n_	$f_{{2{\text{n}}}} = \frac{{{\text{z}}_{1} {\text{z}}_{3} {\text{z}}_{4} }}{{{\text{z}}_{1} + {\text{z}}_{2} {\text{z}}_{4} }}f_{1}$	641.98	495.24	429.75	339.87
First stage meshing frequency *f*_1n_	$f_{{1{\text{n}}}} = \frac{{{\text{z}}_{1} {\text{z}}_{2} {\text{z}}_{4} }}{{{\text{z}}_{1} + {\text{z}}_{2} {\text{z}}_{4} }}f_{1}$	526.75	330.16	396.69	496.73

## Changes in vibration characteristics of reducers caused by different anomalies

During the factory inspection process of the product, there may be non-conforming products caused by processing errors, assembly errors, etc. Most of these non-conforming products can be transformed into qualified products by replacing parts. But if the cause of the nonconformity is judged solely based on the experience of the assembly workers, blindly replacing parts often results in the need for multiple replacements to find the cause of the problem. This brings unnecessary trouble to the assembly qualification rate. Therefore, strictly controlling the factory inspection process and using vibration signals to determine the source of problems can greatly ensure the factory qualification rate and improve assembly production efficiency. To this end, it is necessary to clarify the qualified standards for vibration signals of factory products and distinguish the vibration characteristics caused by various errors.

The author traced and tested hundreds of products from RV reducer manufacturers, including samples of four speed ratios of RV40E. The similar patterns were summarized to derive the universal signal characteristics of the product.

### Normal product qualification standard

In previous tests, the noise detection method was generally used to comprehensively evaluate the operational performance of the product. When the sound of the product run smoothly without sharp or abnormal noises, and the forward and reverse rotation noise was similar, it was judged as qualified. But there were many human factors involved in this method. The criterion for judging abnormal noise solely based on the human ear is prone to deviation. Moreover, it is difficult to determine whether the slight abnormal noise is qualified or not. Therefore, enterprises urgently need to improve their existing evaluation standards. The testing method of vibration signal is adopted for detection.

Due to the limited knowledge level of the operators, in order to propose practical and feasible on-site operation standards, only time-domain amplitudes and time-domain waveform were used for judgment. Operators generally cannot understand frequency domain diagrams, so frequency domain diagrams are not suitable as evaluation criteria. By comparing various types of errors, it was found that the type of error could be directly determined from the time-domain graph alone. In subsequent research, the author will also conduct specific analysis on frequency domain graphs to improve existing judgment criteria. At that time, an expert database may be generated based on these frequency characteristics, and the detection software will be further developed to generate an intelligent judgment interface for easy use by operators.

**Fig. 3:**
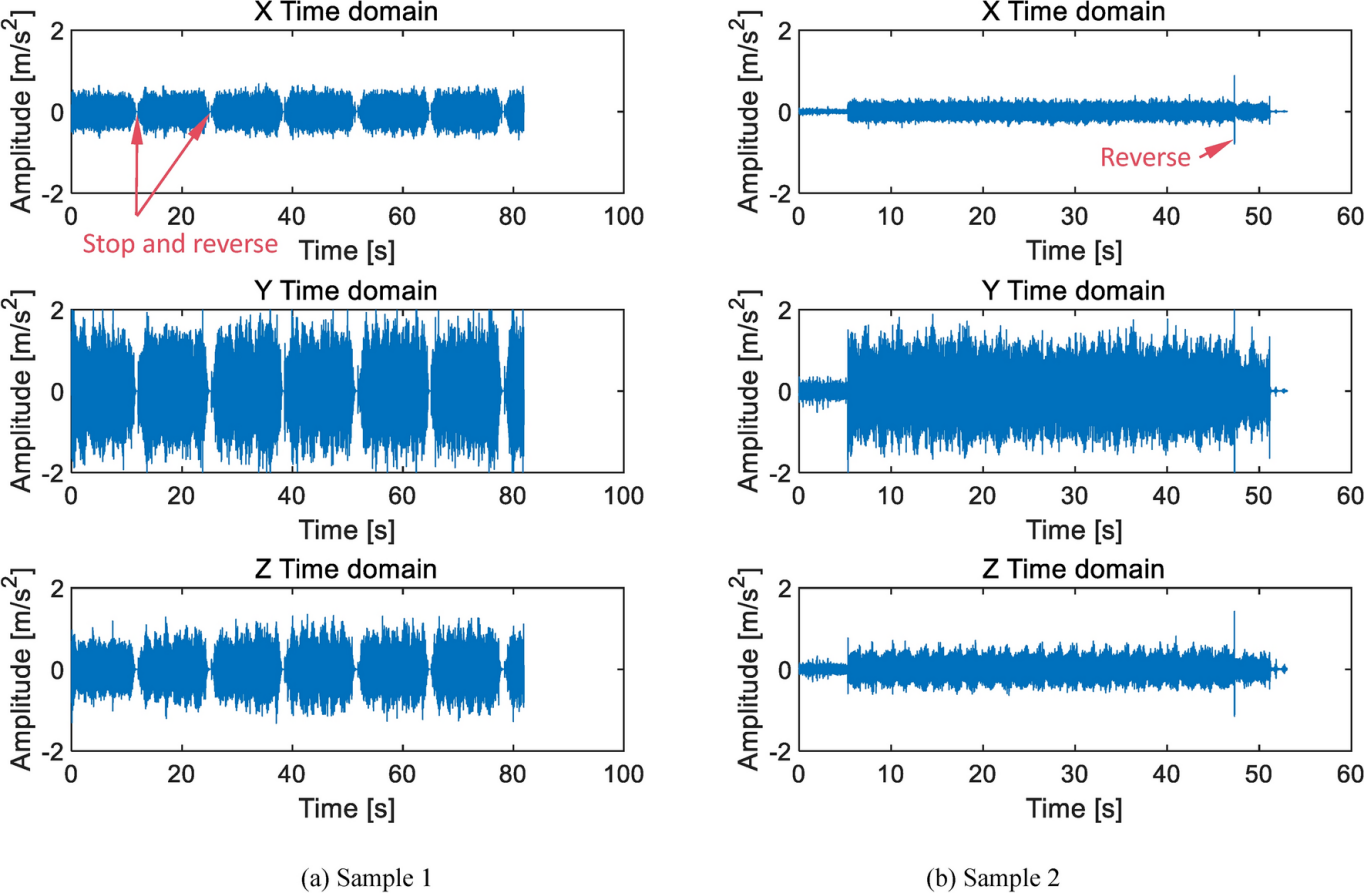
Time domain diagram of qualified products RV40E-121.

The experimental method involved repeated testing in both forward and reverse directions, with the test bench speed set to 2000 rpm as shown in Fig. 1. The time-domain plots of the sample signals under normal condition is shown in Fig. 3. Figure 3a shows a product sample of industry leader Nabtesco. Figure 3b shows the manufacturer’s sample. Both samples were RV40E-121 qualified products with the same structural parameters and materials. The vibration signals of qualified products in the author’s samples were very similar. During testing, due to the workers’ arbitrary operation, it was possible for the forward rotation to stop and then reverse (Fig. 3a), or for the forward rotation to directly reverse (Fig. 3b). Due to the lack of absolute testing standards for this product, an analogy standard for qualified products is set by comparing more than 50 qualified samples. The specific standard is that the time-domain amplitudes in the X, Y, and Z directions of the product are all less than ± 2 m/s^2^, which is considered qualified. The qualified standard of ± 2 m/s^2^ amplitude here does not include the transient amplitude of commutation and starting impact, as well as individual slight impact amplitudes exceeding the standard.

The current testing standard is the vibration testing standard applicable to the product, which was summarized after six months of continuous tracking of the production testing process under the existing technology. Of course, with the continuous optimization of production processes in the later stage, product performance will continue to improve, and this standard will also be correspondingly raised.

As shown in Fig. 3, the amplitude of the normal test signal is relatively stable and balanced. The amplitudes in the three directions are similar. Although there are small cyclical fluctuations, there are no obvious prominent shocks. The characteristic of gear rotation causes small periodic signals, which are inevitable and normal features. But if the amplitude is too large, it indicates abnormal wear or collision. If it exceeds the qualified standard threshold, it is judged as abnormal. There are many factors that cause nonconformity. Below are several types of anomalies that account for a large proportion in actual production.

### Center axis eccentricity

Eccentricity is a common issue encountered in testing. This type of detection sounds like there is a noticeable periodic abnormal noise. Eccentricity may be caused by large end runout of the central axis itself, or by a combination of positional errors of the two cycloid gears and machining errors of the eccentric axis, resulting in the compression of the central axis by the planetary gears.

#### Dimensional chain fit relationship

**Fig. 4:**
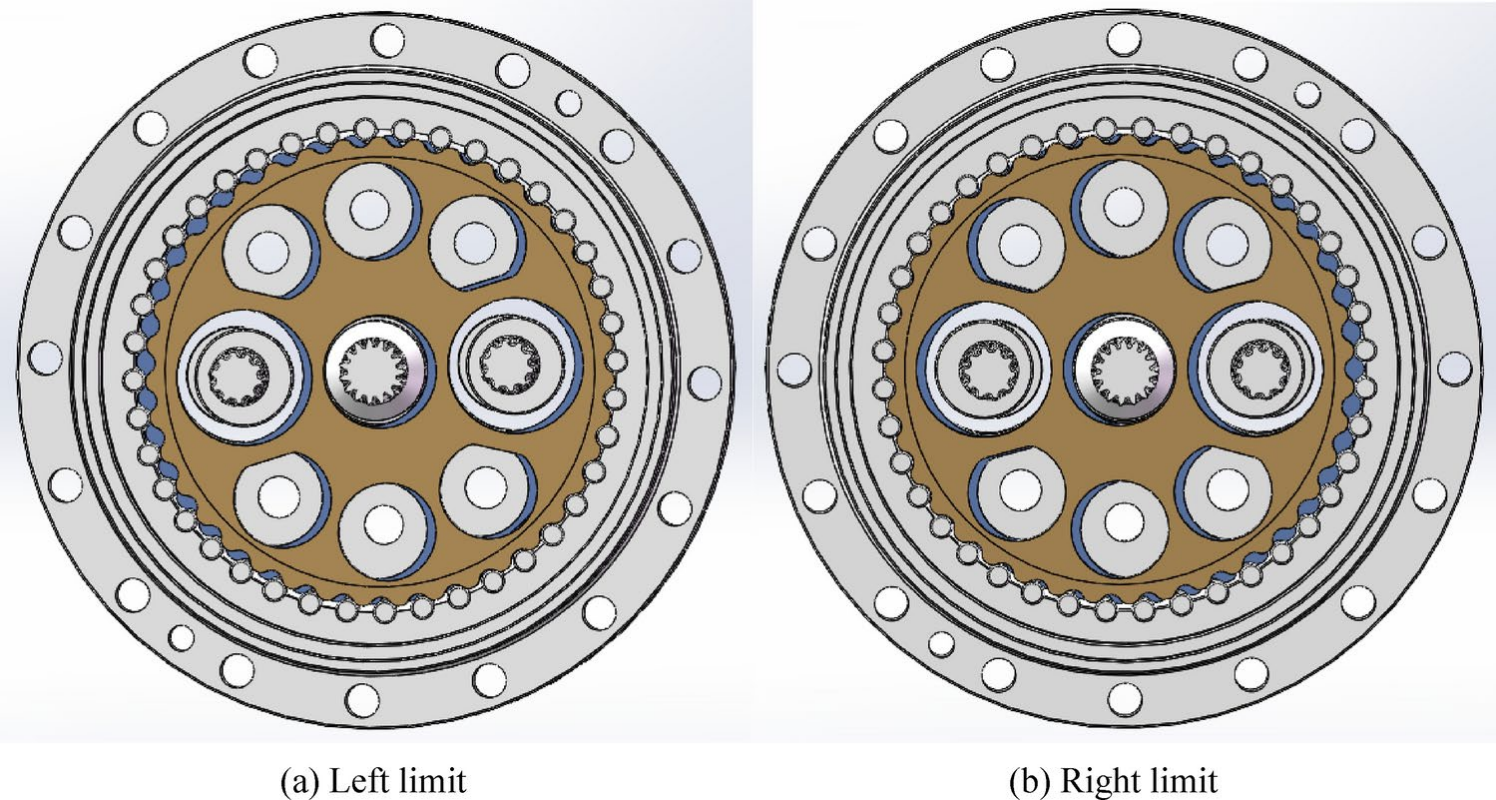
Center axis eccentric left and right limit positions.

When the eccentric axis rotates, it drives the cycloid gear to rotate. The eccentric axis rotates once and the cycloid gear rolls one pin. When the cycloid gear rolls to a position aligned with the two bearing holes, it is the eccentric limit position of the entire dimension chain. When the cycloid gear 2 is on the left side, it is called the left limit, and when it is on the right side, it is called the right limit, as shown in Fig. 4. The eccentricity error of the two extreme positions is different. Therefore, two periodic peaks separated by 180° phase will be generated.

**Fig. 5:**
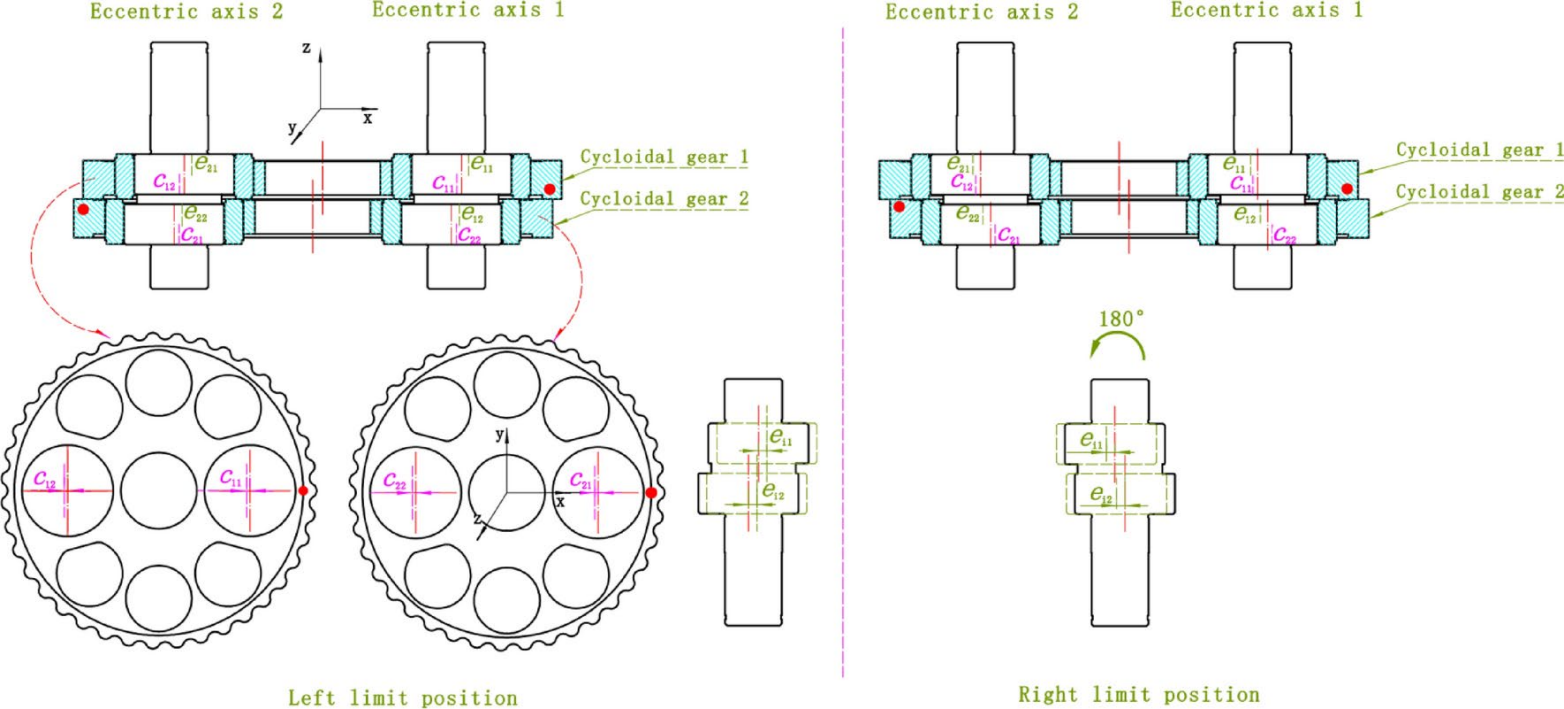
Matching relationship between cycloid gear and eccentric axis dimension chain.

The eccentric axis is matched with the cycloid gear through cylindrical roller bearings. Due to the mass production of cycloid gears, there are often positional deviations in the same direction when machining the two bearing holes on them. The positional deviation of the cycloid gear in the X-direction is defined as *c*_ij_, where *i* is the cycloid gear *i* and *j* is the hole *j* (*i* = 1, 2; *j* = 1, 2), as shown in Fig. 5. Only the X-axis error is discussed here, and the Y-axis error has little impact on the overall limit position calculation, so it is ignored here. To ensure correct positioning during processing, each cycloid gear is marked with a positioning point, as shown by the red dot on the cycloid gear in Fig. 5. During assembly, the positioning surfaces of the two cycloid gears are in contact and stacked at a 180° angle. The upper circle of the eccentric axis is eccentric circle 1, and the lower circle is eccentric circle 2. The positional deviation of the centers of two circles in the x-direction is *e*_ij_, where *i* is the eccentric axis *i* and *j* is the eccentric circle *j* (*i* = 1, 2; *j* = 1, 2).

The left and right limit positions of the dimension chain composed of eccentric axis and cycloid gear are shown in Fig. 5. At the left limit position, the dimensional chain error between eccentric axis 1, 2 and cycloid gear 1 is *A*_11_, the dimensional chain error between eccentric axis 1, 2 and cycloid gear 2 is *A*_12_, and the total error is *A*_13_. The dimension chain relationship here is

1 $$\left\{ {\begin{array}{*{20}c} {A_{11} = \left( {e_{11} - c_{11} } \right) - \left( {e_{21} - c_{12} } \right)} \\ {A_{12} = \left( {c_{21} - e_{12} } \right) - \left( {c_{22} - e_{22} } \right)} \\ {A_{13} = A_{11} - A_{12} } \\ \end{array} } \right.$$

At the right limit position, the dimensional chain error between eccentric axis 1, 2 and cycloid gear 1 is *A*_21_, the dimensional chain error between eccentric axis 1, 2 and cycloid gear 2 is *A*_22_, and the total error is *A*_23_. At this point, the error of the cycloid gear remains unchanged, while the direction of the error of the eccentric axis changes due to a 180° rotation. The dimension chain relationship here is

2 $$\left\{ {\begin{array}{*{20}c} {A_{21} = \left( { - e_{11} - c_{11} } \right) - \left( { - e_{21} - c_{12} } \right)} \\ {A_{22} = \left( {c_{21} + e_{12} } \right) - \left( {c_{22} + e_{22} } \right)} \\ {A_{23} = A_{21} - A_{22} } \\ \end{array} } \right.$$

Due to the difference in left and right limit errors, there are two periodic peaks when the cycloid gear rotates once. The period with higher amplitude is ${\text{max}}\left[ {\left| {A_{13} } \right|,\left| {A_{23} } \right|} \right]$. The error of the dimension chain needs to be limited within a certain range.

3 $$\left\{ {\begin{array}{*{20}c} {A_{11} \in \left[ {l_{1} ,u_{1} } \right]} \\ {A_{12} \in \left[ {l_{2} ,u_{2} } \right]} \\ {A_{21} \in \left[ {l_{1} ,u_{1} } \right]} \\ {A_{22} \in \left[ {l_{2} ,u_{2} } \right]} \\ {A_{min} \le A_{13} ,A_{23} \le A_{max} } \\ \end{array} } \right.$$

In the formula, *l*_1_ and *l*_2_ are the lower limits of the size chain; *u*_1_ and *u*_2_ are the upper limits of the size chain; *A*_max_ is the limit value of tensile elastic deformation for the overall size chain; *A*_min_ is the compression elastic deformation limit value of the total size chain.

When the total error of the dimension chain exceeds the elastic deformation limit, the transmission chain will experience a stuck phenomenon. At this point, a larger torque is required to overcome the stagnation point. When the deviation is too large, there will be a stuck phenomenon.

#### Eccentric fault diagnosis

**Fig. 6:**
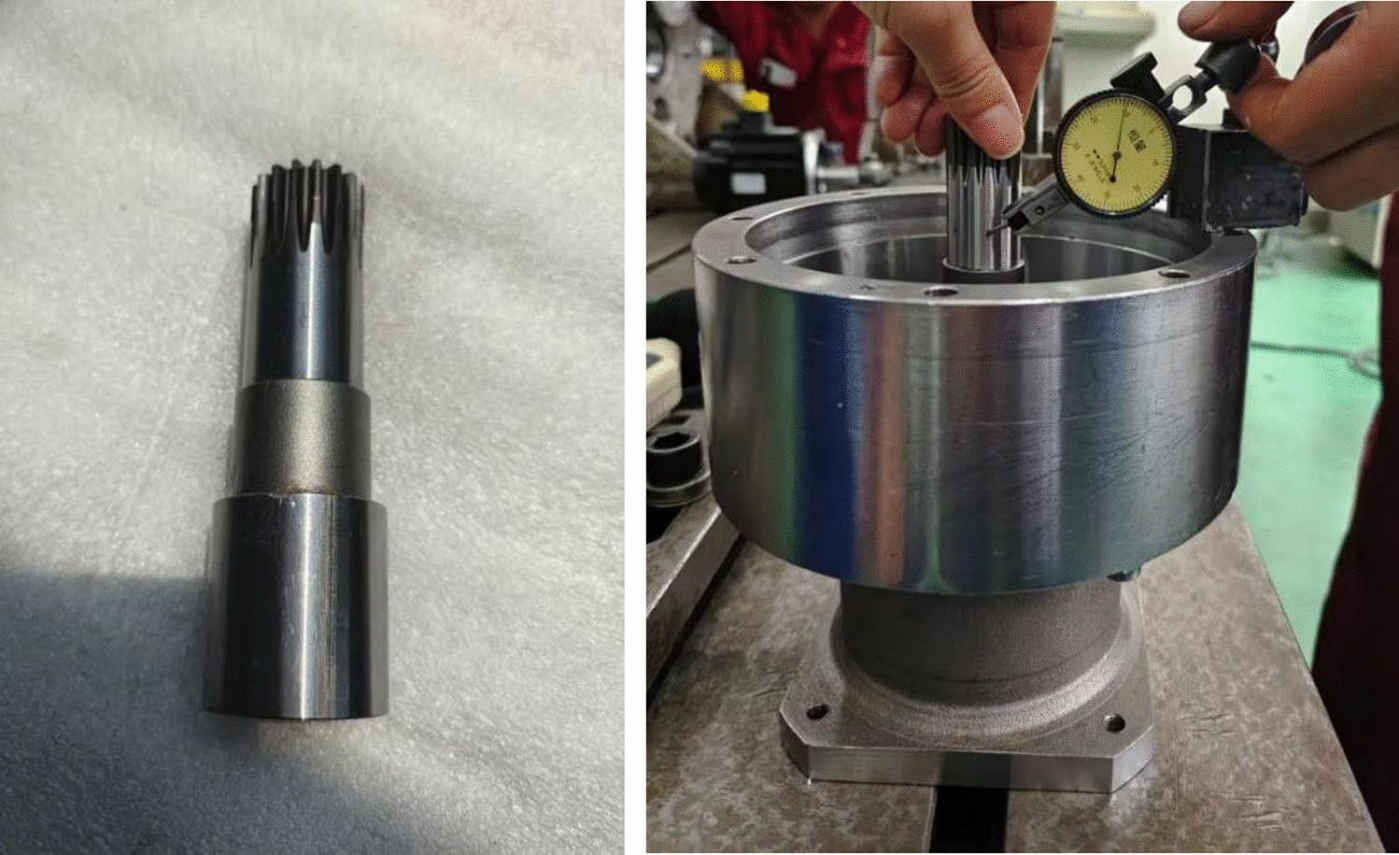
Center axis jumping detection.

**Fig. 7:**
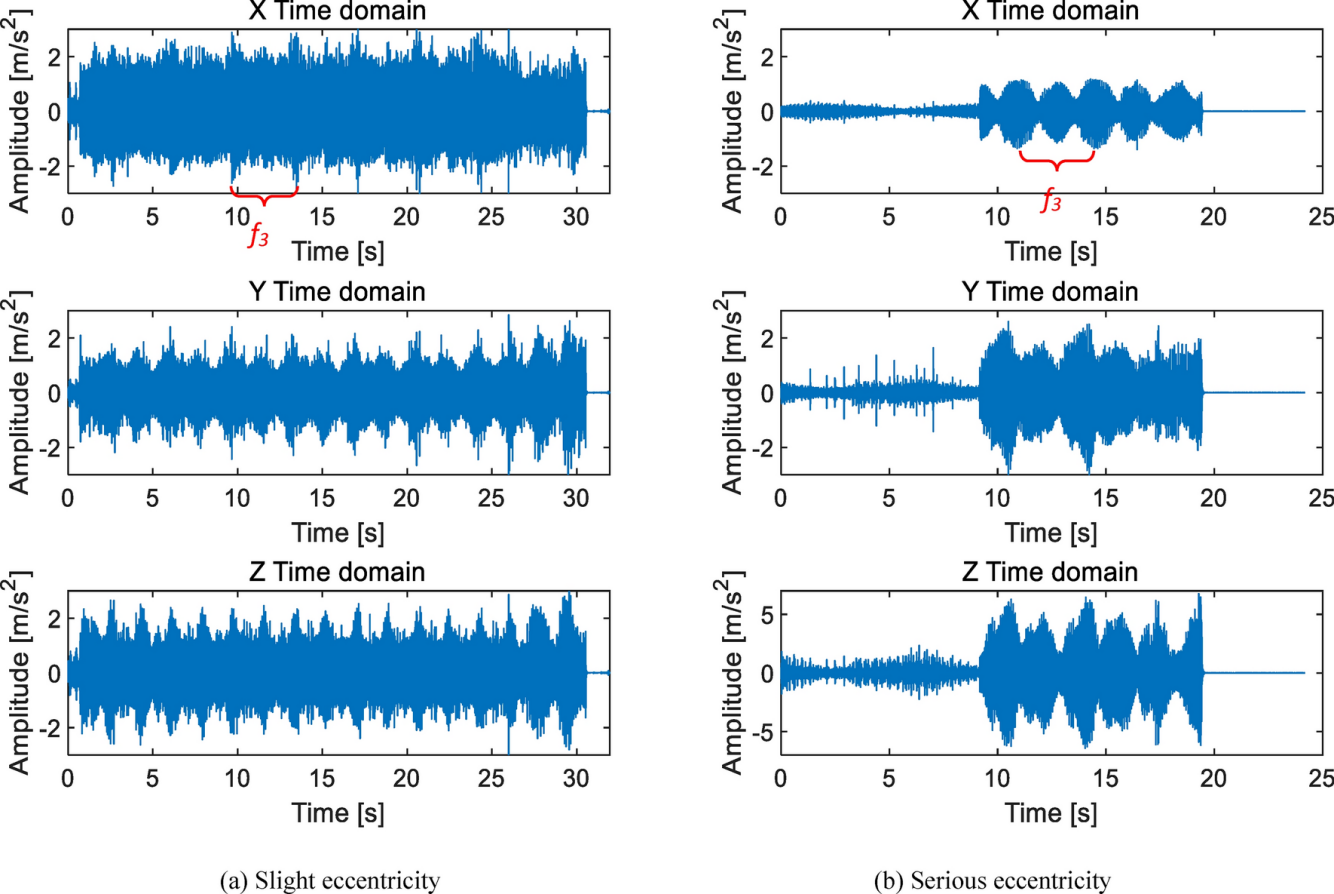
Time domain diagram of the end jumping of the central axis itself RV40E-121.

During the machining process, the central axis may experience significant end jumping due to machining errors. The excircle at the end of the central axis generally does not have significant machining errors. However, in the later processing of slot holes that match the motor shaft, the positional and vertical errors of the slot holes will be amplified by the end after installation. The jumping of the excircle surface of the central axis was detected, as shown in Fig. 6. The larger the excircle jumping, the more obvious the periodic abnormal noise. When the end jumping exceeds 0.01 mm, it would cause significant periodic abnormal noise, as shown in Fig. 7.

As shown in Fig. 7, the eccentricity caused by the end jumping of the central axis have two cycles of high and low. Two cycles have the same frequency and a phase difference of 180°. This is the stagnation impact point at the left and right extreme positions shown in Fig. 5. Figure 7a shows slight eccentricity, periodic impact can be detected, and the amplitude slightly exceeds the qualified standard ± 2 m/s^2^. Figure 7b shows serious eccentricity, with the Z-axis amplitude exceeding the normal amplitude by more than 1 time. The characteristic frequency is the rotational frequency *f*_3_ of the cycloid gear. When the speed ratio is 121, the characteristic frequency is *f*_3_ = 0.28 Hz, which means the period *T* = 3.63 s. This feature is clearly visible and can serve as a basis for judgment.

**Fig. 8:**
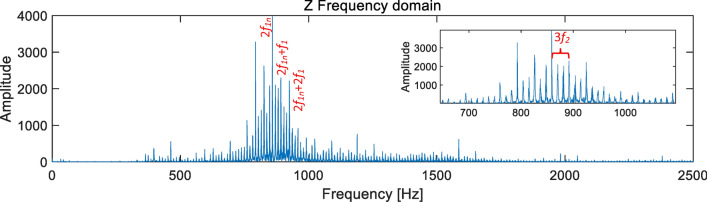
Frequency domain diagram of the end jumping of the central axis itself RV40E-121.

In the frequency domain diagram of the sample in Fig. 7b, it can be clearly seen that the main peak value caused by the left and right limits is the second harmonic of the first-order meshing frequency 2*f*_1n_, the side frequency *f*_1_ = 33.33 Hz represents the fault characteristics of the central axis, and the high-order eccentric axis rotation frequency *f*_2_ = 11.02 Hz, as shown in Fig. 8.

**Fig. 9:**
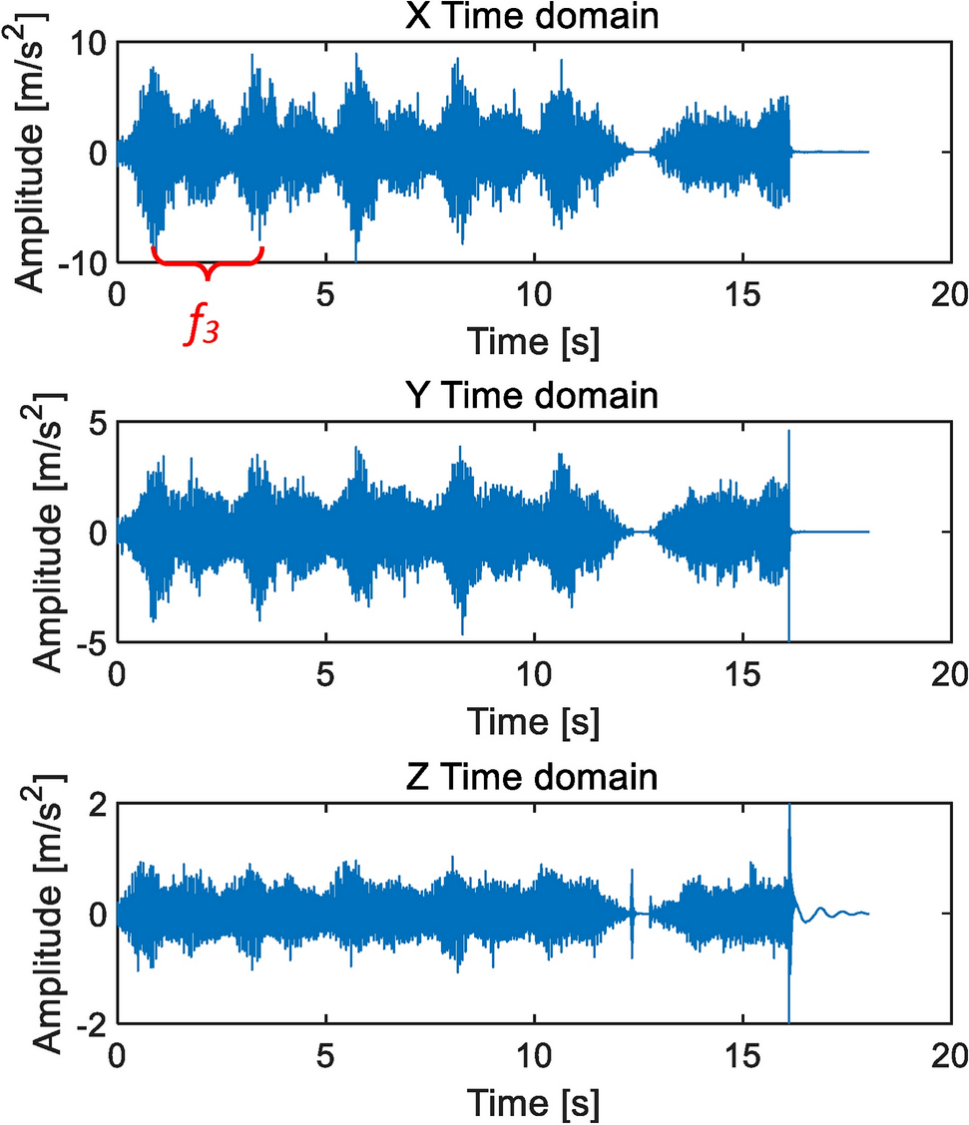
Time domain diagram of planetary gear compression center axis RV40E-105.

In the transmission chain composed of planetary carriers, eccentric axiss, and cycloidal gears, the accumulated machining errors of several parts will also compress the central axis at the end. The eccentricity caused by this compression is slightly different from the eccentricity of the central axis itself. In addition to the periods in the frequency spectrum at the left and right limit positions of the eccentric axis, there will also be noticeable abnormal noise. The noise is caused by the cycloidal gear transmission chain reaching its limit position and forcefully passing through the limit position due to insufficient size. The time-domain diagram at this time is shown in Fig. 9.

As shown in Fig. 9, the effects of the two types of eccentric faults are almost identical, with *f*_3_ as the characteristic frequency and periodic vibrations occurring at the left and right limit positions.

### First stage planetary gear defect

#### Forward and reverse rotation noise difference

**Fig. 10:**
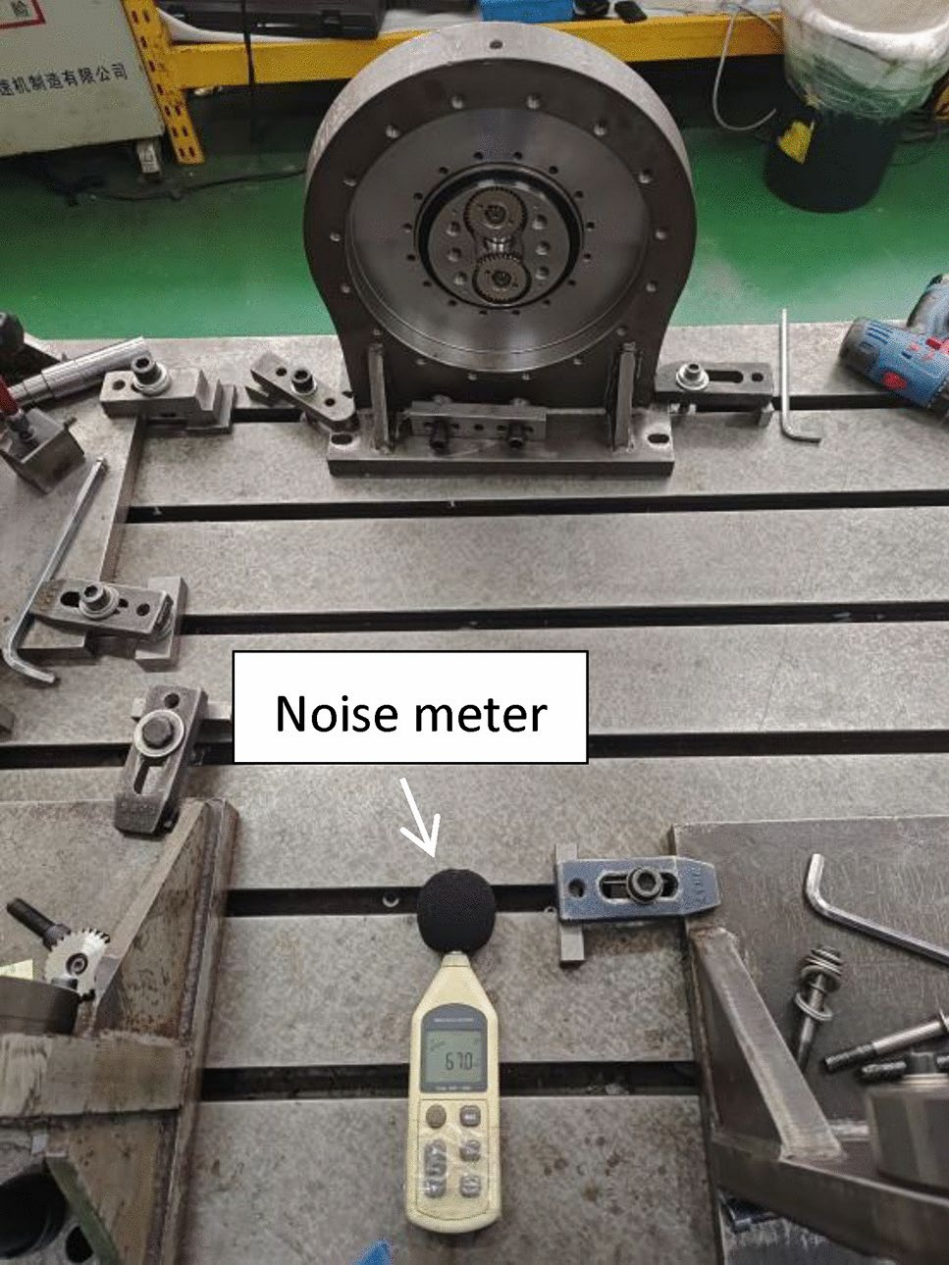
Noise Test.

A common type of detection is the presence of forward and reverse rotation noise with a difference. The testing method is shown in Fig. 10. It rotated forward at a speed of 2000 r/min for 5–10 s, then stopped, and reversed for another 5–10 s. The decibel values of noise during both forward and reverse rotation were measured. The noise values and noise differences are important indicators for factory inspection. Most assembly issues and processing defects would be reflected in the noise. The noise difference often exists during factory inspection. Through extensive comparison of factory products and customer after-sales feedback. The factory standard is defined as when the noise difference is greater than 3db, the product is judged as unqualified.

**Fig. 11:**
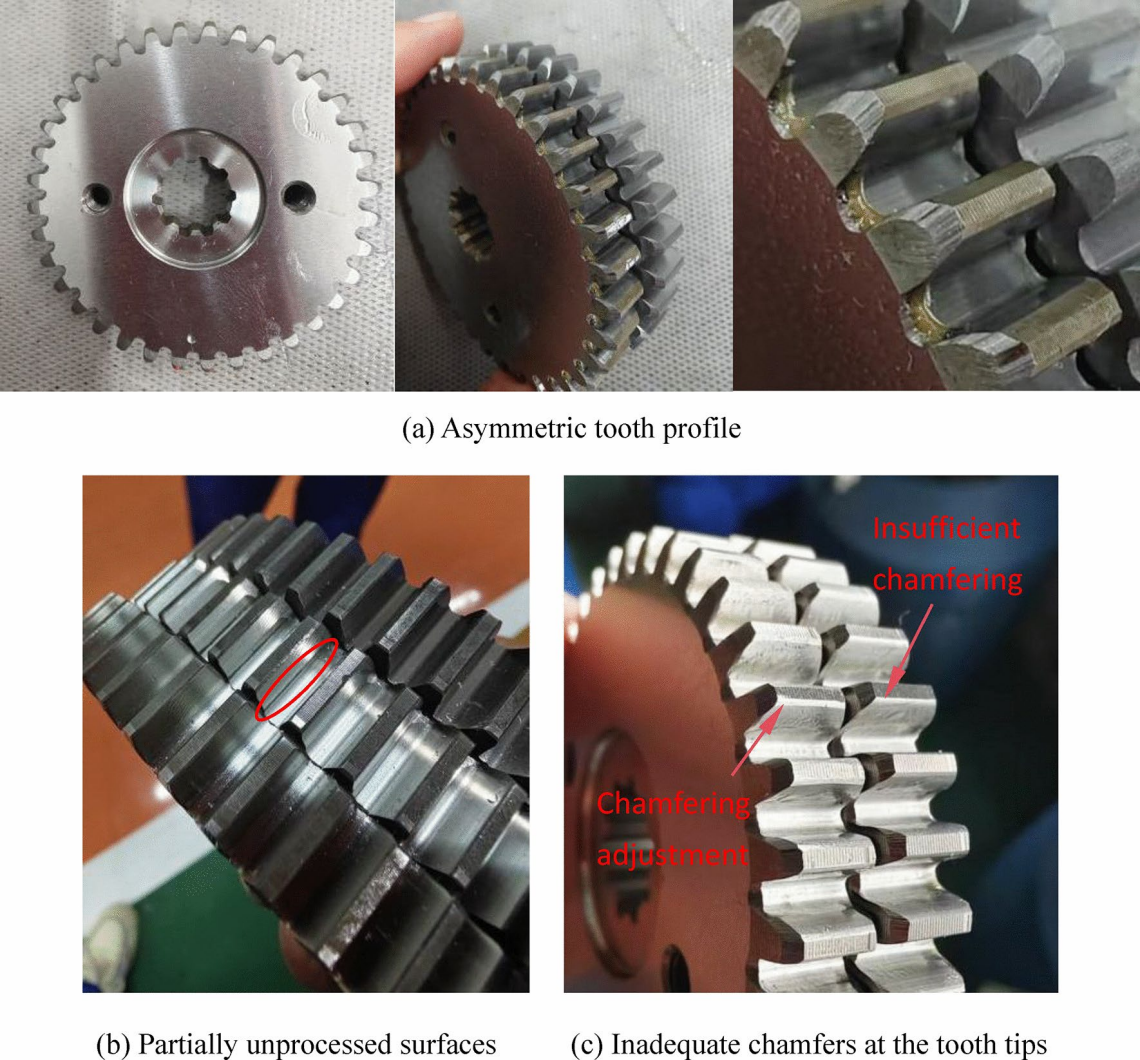
Asymmetry of the first stage planetary gear tooth surface.

**Fig. 12:**
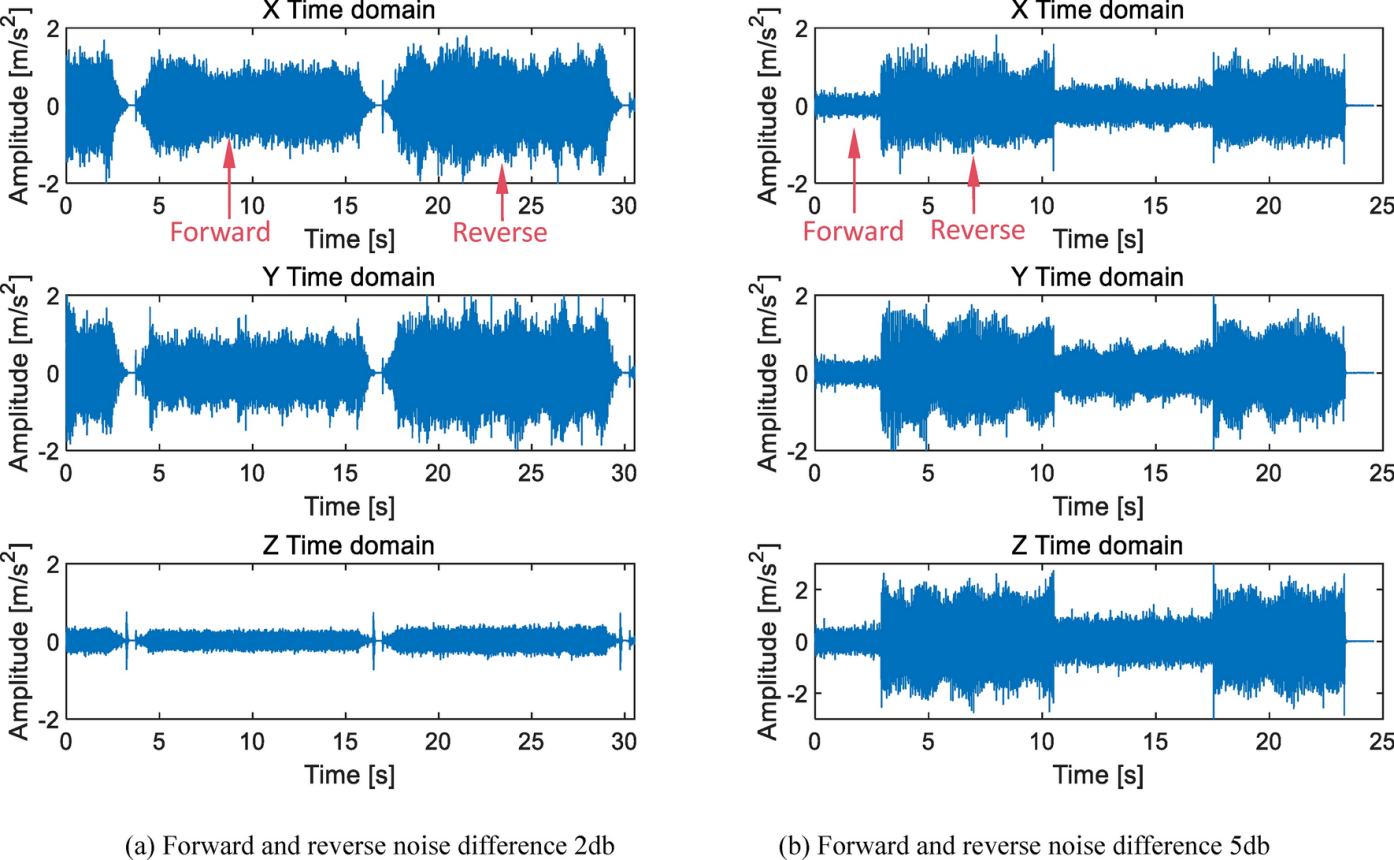
Time domain diagram of forward and reverse noise difference.

The test parts in this article are all actual application parts in the factory. Gears with minor scratches in actual production may pass production inspections and flow into assembly processes. During the factory inspection, it was found that there were noise differences, abnormal noises, and abnormal vibration signals in the finished product. During the secondary repair and inspection of such products, it was found that most of the problems were caused by the first stage planetary gear. From a large number of samples, it was known that the causes of excessive noise values and noise differences were mostly due to the asymmetry of the first stage planetary gear tooth surface, which might be caused by problems such as asymmetric tooth profile, partially unprocessed surfaces, and inadequate chamfers at the tooth tips, as shown in Fig. 11. There are many sources of faults that cannot be directly determined from the time domain, but the amplitude difference in the time domain can be used to determine whether they are qualified or not, which serves as the on-site inspection standard. After comparing a large number of samples, it was concluded that when the forward and reverse rotation amplitudes differ by 0.6 m/s^2^, the noise difference is approximately 1db; When the amplitude difference between forward and reverse is 1 m/s^2^, the noise difference is 2db; When the amplitude difference between forward and reverse is 2 m/s^2^, the noise difference is 5db, as shown in Fig. 12.

In Fig. 12a, the amplitude difference between forward and reverse rotation is 1 m/s^2^, and the noise difference is 2db. Due to the overall amplitude being less than ± 2 m/s^2^, a noise difference of 2db is acceptable, therefore it can be judged as qualified. In Fig. 12b, the amplitude difference between forward and reverse rotation is 2 m/s^2^, and the noise difference is 5db. Although the overall amplitude is also less than ± 2 m/s^2^, it is judged as unqualified. The reason for the difference in noise between forward and reverse rotation is generally due to the substandard machining quality of the first stage planetary gear. This problem can generally be solved by replacing the planetary gear.

It should be noted that lubricants can also have an impact on vibration. Normally, we first detect the noise when no lubricant is added. At this moment, the abnormal noise is quite noticeable. When lubricant is added to the reducer, the noise generally decreases by about 5db-10db. Abnormal noises are not easily detected. And after injecting lubricant, splashing occur during testing, so lubricating grease is usually injected only after the reducer is judged to be qualified.

#### Surface defects on planetary gears

**Fig. 13:**
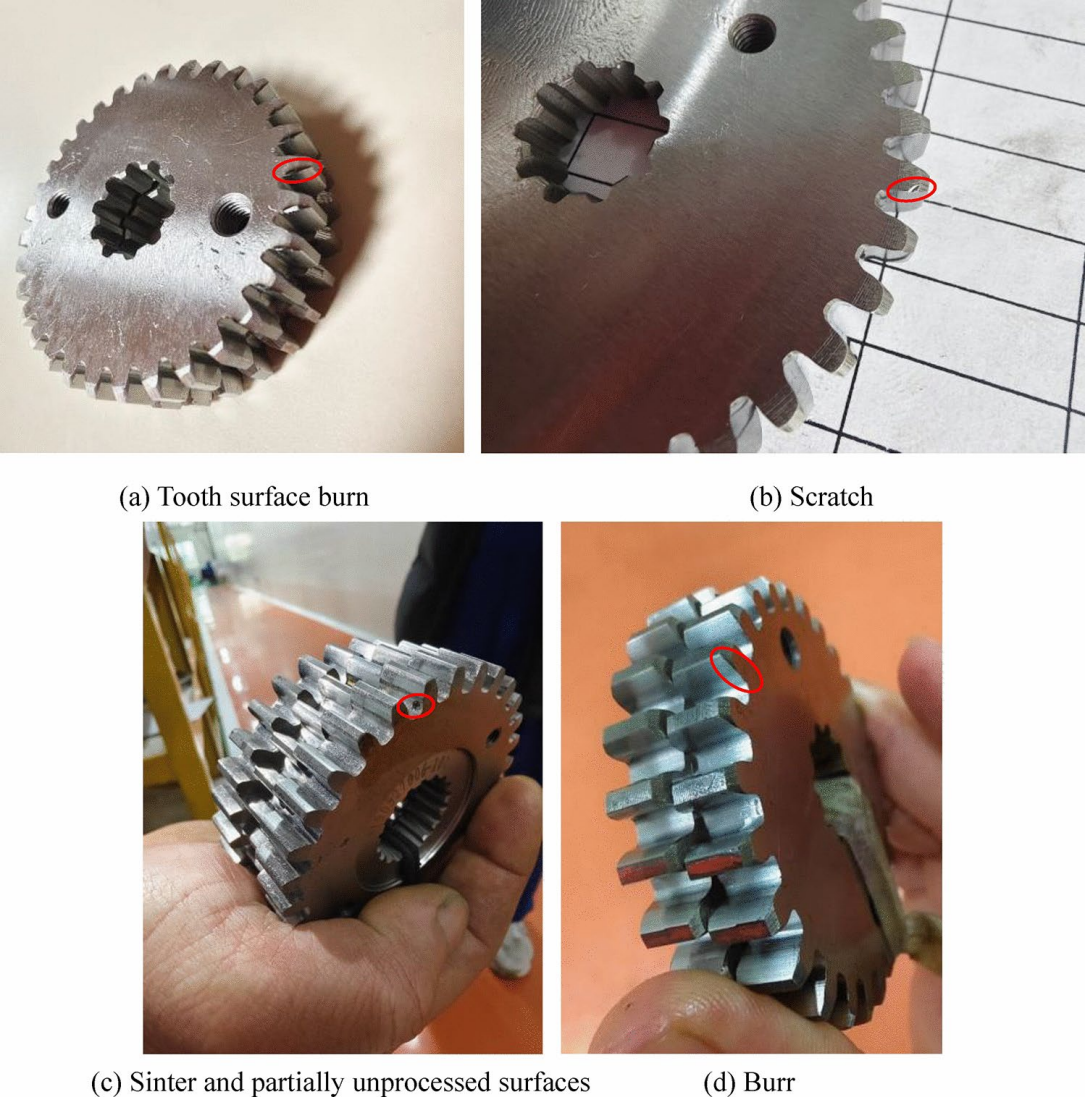
Gear surface defects.

**Fig. 14:**
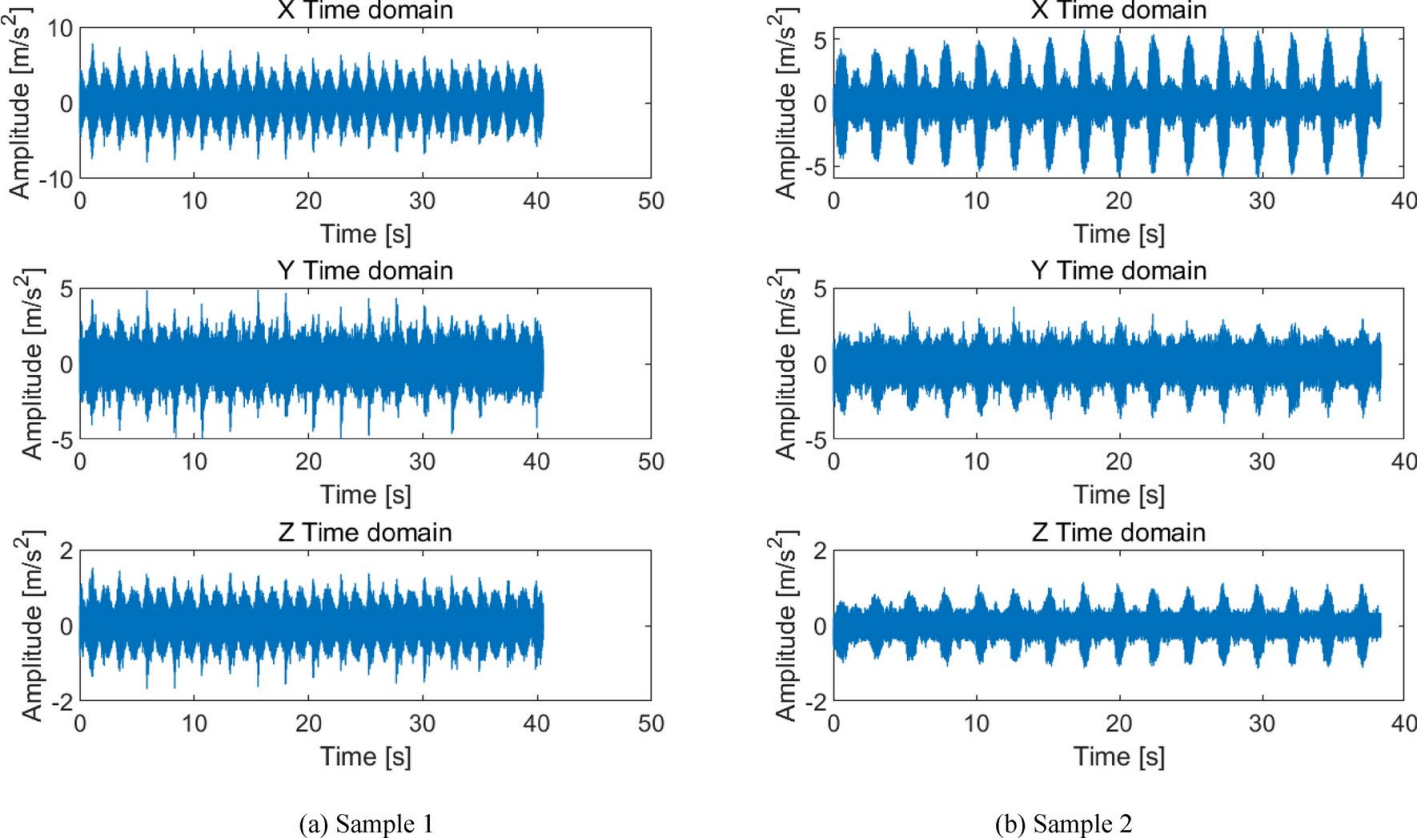
Time domain diagram of gear surface defects RV40E-81.

The noise difference is generally caused by defects in all teeth of the first stage planetary gear, and the vibration frequency is the first stage meshing frequency *f*_1n_. Due to the meshing frequency being in the mid frequency range, there is no impact phenomenon in the time-domain graph. When individual teeth of the planetary gear have defects, the fault characteristic frequency is not *f*_1n_, but the planetary gear rotation frequency *f*_3_. The common surface defects of planetary gears mainly include scratches, tooth surface burns, burrs, and other machining problems, as shown in Fig. 13. There would be obvious periodic collision sounds when listening to sound. Although the fault characteristics of surface defects on planetary gears are the same as those of eccentricity on the central axis. However, due to the fact that the planetary gear is located outside the reducer and close to the sensor, surface defects on the planetary gear are reflected as stable equal amplitude impacts in the time-domain signal. The impact amplitude is extremely obvious and regular, as shown in Fig. 14.

As shown in Fig. 14, the surface defect of the first stage planetary gear is a hard point during meshing, and the resulting impact is a short-term transient large impact. This is particularly evident in the X-direction time-domain plot of Fig. 14. This fault can be resolved by replacing the gear.

### The fixture is not locked tightly

**Fig. 15:**
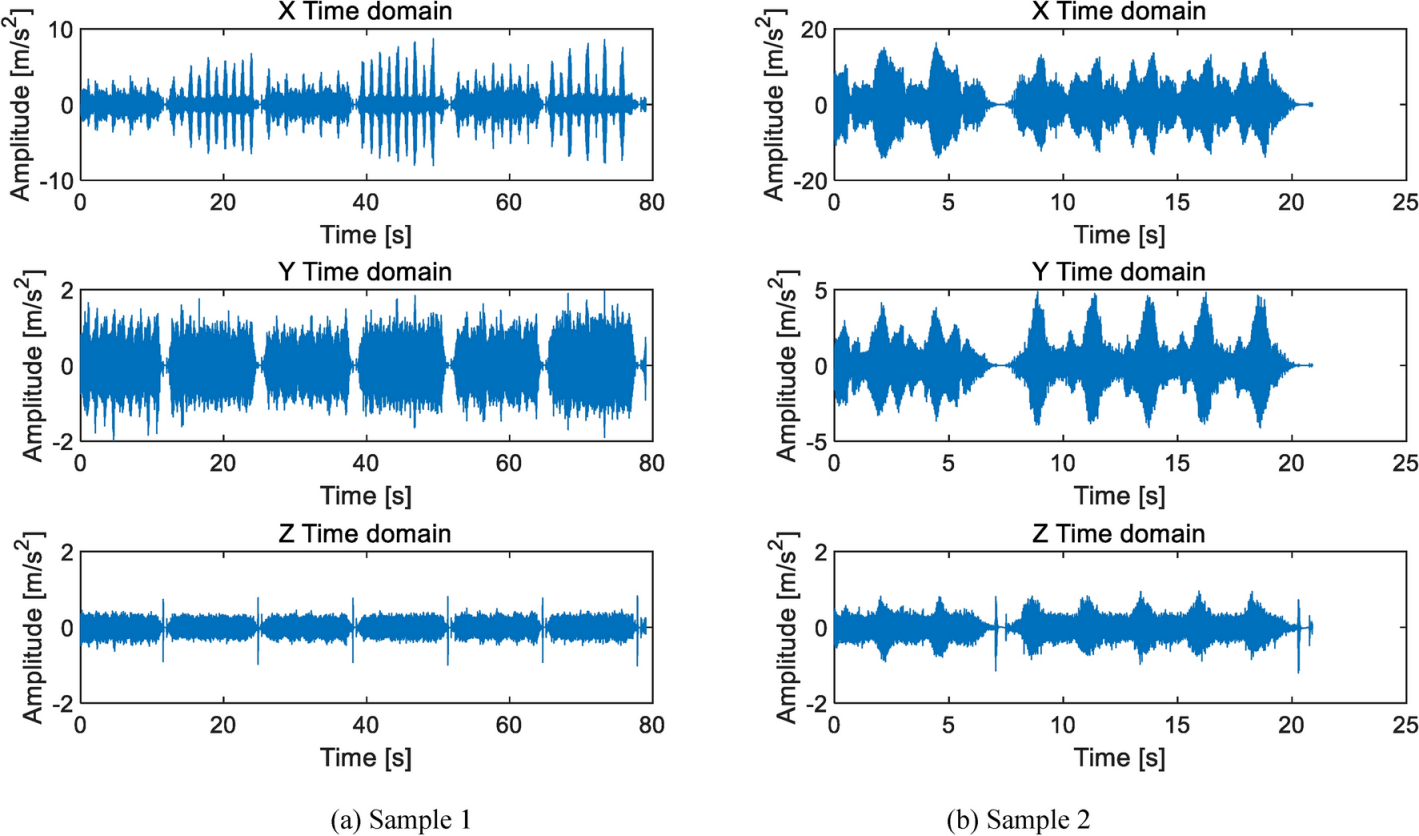
The fixture is not locked tightly.

There are also some human operation errors during the testing process, one of which is that the fixture was not locked tightly during the inspection. At this time, the time-domain characteristics show that the amplitude of the X-axis is much larger than that of the Y-axis. Sometimes it also presents short-term and multiple large amplitude impacts like fish bones. As shown in Fig. 15.

As shown in Fig. 15, when the fixture is not locked tightly, the tested product will move slightly in the X and Y planes. Due to gravity, the Y-direction movement is slightly weaker. The X-direction moves freely under insufficient constraints, and its vibration frequency is not constant. Figure 15 shows the time-domain signals of two samples when they are not locked. The impact frequency in the X direction of the two is not the same, which is related to the degree of locking tightly. When the lock is loose, there is a gap between the product and the fixture, which can quickly swing. When the lock is slightly tightened, the rigidity is relatively high. There is not as much clearance margin, and the vibration frequency is relatively low. But compared to normal vibration, the lack of locking ultimately leaves a gap for vibration, with vibration amplitudes far exceeding normal values and more prominent in the X-direction. When this situation occurs, the fixture needs to be locked and remeasured.

### Statistical analysis

The aforementioned time-domain features still have errors in manual detection due to human judgment. Therefore, the author quantifies these fault features from a statistical perspective as a supplementary basis for determining anomalies. The root mean square (RMS), commonly used in vibration measurement, is adopted to quantitatively evaluate the overall signal. The RMS value can measure the energy of the vibrating object and also provides information about the intensity of the object’s motion. It allows for quantitative control of the overall amplitude of the signal. When any component of X, Y, or Z is more than twice the standard value, it is considered that the product has potential faults and should be judged as a defective product for repair. The addition of this indicator makes the judgment more comprehensive and visible. All samples in the text are summarized, and their RMS values are listed in Table 3 below.

**Table 3:** Root mean square statistical analysis of the samples in the paper.

Figure number	Root mean square					
	X	ratio	Y	ratio	Z	ratio
Figure 3a	**0.1387**	**1**	**0.3885**	**1**	**0.2099**	**1**
Figure 3b	0.0660	0.48	0.3021	0.78	0.1122	0.53
Figure 7a	0.6433	4.64	0.3781	0.97	0.5250	2.50
Figure 7b	0.1998	1.44	0.3087	0.79	0.8715	4.15
Figure 9	0.9646	6.95	0.4841	1.25	0.1792	0.85
Figure 12a	0.3599	2.59	0.3571	0.92	0.1019	0.49
Figure 12b	0.2515	1.81	0.2939	0.76	0.4903	2.34
Figure 14a	1.1773	8.49	0.6623	1.70	0.2489	1.19
Figure 14b	0.8802	6.35	0.6042	1.56	0.1855	0.88
Figure 15a	0.6155	4.44	0.2452	0.63	0.1017	0.48
Figure 15b	2.0424	14.73	0.4643	1.20	0.1424	0.68

Significant values are in (bold).

In Table 3, Fig. 3 represents the normal samples. Clearly, the performance in Fig. 3b is slightly better. Here, the values in the X, Y, and Z directions of Fig. 3a are used as the standard values, that is, (0.1387, 0.3885, 0.2099) are taken as the standard samples for the root mean square in the text. In actual production, the value will be revised by comparing a large number of samples to provide a reasonable upper limit for the analogous standard. Here, Fig. 3a is taken as the standard sample, and the values of each component of the other samples are compared with the values of Fig. 3a. The resulting ratios are listed to the right of the components.

From Table 3, it can be seen that all faulty samples have at least one component ratio that is more than twice the standard value. Among all the ratios that exceed the standard, the X-direction is the most significant, followed by the Z-direction. For instance, the central axis eccentricity fault in Fig. 7a,b has components that are four times the standard value. In Fig. 9, the X-direction is 6.95 times the standard value. The noise difference fault in Fig. 11 is twice the standard value. Surface defect fault characteristics are quite evident, with Fig. 13a having an X-direction ratio of 8.49 and Fig. 13b an X-direction ratio of 6.35. The work fixture not being tightened is evident in Fig. 14a with an X-direction ratio of 4.44 and Fig. 14b with an X-direction ratio of 14.73. Therefore, it is clear that the root mean square can indeed reflect the performance level of the product to a certain extent and can be used as an auxiliary indicator to set the inspection standard for determining factory qualification.

## Conclusion

This study conducted vibration characteristic tests on RV40E reducers at different speed ratios. Through a large number of sample comparisons, suitable vibration characteristic factory testing standards for production enterprises were provided. Detailed on-site inspection standards have been developed based on research and analysis results for defective products such as eccentricity, gear defects, and detection errors. This standard can cover 90% of defect types within the factory. The sample is specific and executable. This study solves the problem of the lack of clear vibration detection standards in the industry. For the first time, such a detailed sample of internal data within a company has been provided. It has extremely practical engineering application value. It provide data reference for the development of RV reducer industry.

The samples provided in this study were selected from some easily distinguishable features. There are still some ambiguous features at the edge of the qualified line in practical applications of factory inspection. The author will continue to explore frequency characteristics as a supplementary basis in future research.

In the next step, the author will use machine learning to automatically classify fault categories. Based on sample data, machine learning methods will be used to develop a vibration testing software for the factory inspection of RV reducers. This software will be able to distinguish between qualified and defective products through vibration testing. It will also be able to identify the type and location of defects in defective products, guiding assembly workers to carry out precise repairs and improving repair efficiency. After further supplementing the sample features of different fault degrees under the same fault type, the system will be upgraded to provide not only the type of fault but also more detailed information about the degree of the fault.

In production and processing, different process parameters can affect overall performance. Due to the coupling of multiple parameters, this impact is not easily determined directly. In the later stage, further tracking of changes in product vibration characteristics under different process routes will be conducted. By improving the process routes and using vibration testing methods, vibration performance indicators for different error parameters of the assembly parts will be provided. This will help in selecting better process schemes, thereby guiding product process improvements and production processes, ultimately enhancing product quality.

The criteria established by this study have been initially implemented in the company’s trial production and will continue to be refined in the subsequent mass production. During the production process, new samples will be continuously supplemented and improved, and ultimately the vibration characteristic detection step will be integrated into the product’s automated batch testing assembly line, thereby achieving the company’s automated production.

## Supplementary Information

Supplementary Information.

## Data Availability

The authors declare that the data supporting the findings of this study are available within the paper and its Supplementary Information files.

## List of symbols

*f*_1_: Central axis rotation frequency
*f*_2_: Clanetary gear rotation frequency
*f*_3_: Cycloid gear rotation frequency
*f*_1n_: First stage meshing frequency
*f*_2n_: Second stage meshing frequency
*v*_1_: Input axis rotation speed
*z*_1_: Number of teeth on central axis
*z*_2_: Number of teeth on planetary gear
*z*_3_: Number of teeth on cycloid gear
*z*_4_: Number of teeth on pin wheel housing
*r*: Speed ratio
*A*_11_: At the left limit position, the dimensional chain error between eccentric axis 1, 2 and cycloid gear 1
*A*_12_: At the left limit positionthe, the dimensional chain error between eccentric axis 1, 2 and cycloid gear 2
*A*_13_: The total error, A_13_ = A_11_–A_12_
*c*_ij_: Position deviation of the jth hole in the x-direction of the cycloid gear *i* (*i* = 1,2; *j* = 1, 2)
*e*_ij_: Position deviation of eccentric circle j in the x-direction on eccentric axis *i* (*i* = 1,2; *j* = 1, 2)
*A*_21_: At the right limit position, the dimensional chain error between eccentric axis 1, 2 and cycloid gear 1
*A*_22_: At the right limit position, the dimensional chain error between eccentric axis 1, 2 and cycloid gear 2
*A*_23_: The total error, A_23_ = A_21_–A_22_
*l*_1_: Lower limit of size chains* A*_11_ and *A*_21_
*l*_2_: Lower limit of size chains *A*_12_ and *A*_22_
*u*_1_: Upper limit of the size chains *A*_11_ and *A*_21_
*u*_2_: Upper limit of the size chains *A*_12_ and *A*_22_
*A*_min_: Compression elastic deformation limit value of the total size chain
*A*_max_: Limit value of tensile elastic deformation for the overall size chain
*T*: Period

## References

[1] Wang, Z. et al. Effect of multifactor interaction on the accuracy of RV reducers. *Int. J. Rotat. Mach.* **2023**(1), 5692229 (2023).

[2] Hu, Y., Li, G., Zhu, W. & Cui, J. An elastic transmission error compensation method for rotary vector speed reducers based on error sensitivity analysis. *Appl. Sci.* **10**(2), 481 (2020).

[3] Cheng, W. A calculation method of transmission efficiency for RV reducer. *J. Eng. Res.* **9**(4B), 281–295 (2021).

[4] Wang, H., Fang, K., Li, J. & Xi, C. Analysis and experimental study on vibration characteristics of the RV reducer. *Adv. Mech. Eng.* **15**(6), 16878132231181328 (2023).

[5] Han, Z. et al. Study on nonlinear dynamic characteristics of RV reducer transmission system. *Energies* **17**(5), 1178 (2024).

[6] Zhang, D., Li, X. A., Yang, M., Wang, F. & Han, X. Non-random vibration analysis of rotate vector reducer. *J. Sound Vib.* **542**, 117380 (2023).

[7] Song, W., Tan Jing, Gu. & Jingjun, H. D. Study on torsional vibration of RV reducer based on time-varying stiffness. *J. Vib. Eng. Technol.* **9**(1), 73–84 (2021).

[8] Li, X., Huang, J., Ding, C., Guo, R. & Niu, W. Dynamic modeling and analysis of an RV reducer considering tooth profile modifications and errors. *Machines* **11**(6), 626 (2023).

[9] Wang, H., Zhang, X., Fang, K., Kou, Y. & Zhang, Z. Return error simulation analysis and experimental study for RV reducer with ADAMS. *J. Adv. Mech. Des. Syst. Manuf.* **18**(2), JAMDSM0023–JAMDSM0023 (2024).

[10] Ahn, H. J., Choi, B. M., Lee, Y. H. & Pham, A. D. Impact analysis of tolerance and contact friction on a RV reducer using FE method. *Int. J. Precis. Eng. Manuf.* **22**(7), 1285–1292 (2021).

[11] Qiao, Y., Wang, H., Cao, J. & Lei, Y. Sound-vibration spectrogram fusion method for diagnosis of RV reducers in industrial robots. *Mech. Syst. Sign. Process.* **214**, 111411 (2024).

[12] Raouf, I., Lee, H. & Kim, H. S. Mechanical fault detection based on machine learning for robotic RV reducer using electrical current signature analysis: a data-driven approach. *J. Comput. Des. Eng.* **9**(2), 417–433 (2022).

[13] Tu, Z., Gao, L., Wu, X., Liu, Y. & Zhao, Z. Rotate vector reducer fault diagnosis model Based on EEMD-MPA-KELM. *Appl. Sci.* **13**(7), 4476 (2023).

[14] Jiang, K., Zhang, C., Wei, B., Li, Z. & Kochan, O. Fault diagnosis of RV reducer based on denoising time–frequency attention neural network. *Expert Syst. Appl.* **238**, 121762 (2024).

[15] Chu, X., Xu, H., Wu, X., Tao, J. & Shao, G. The method of selective assembly for the RV reducer based on genetic algorithm. *Proc. Inst. Mech. Eng. Part C: J. Mech. Eng. Sci.* **232**(6), 921–929 (2018).

[16] Jingjun, Gu. et al. Manufacturing quality assurance for a rotate vector reducer with vibration technology. *J. Mech. Sci. Technol.* **33**(5), 1995–2001 (2019).

[17] Yunhai, Y., Guo, Yu. & Xiaoqin, L. Tooth root crack detection of planet gear in industrial robot RV reducer. *Measure. Control* **56**(9–10), 1720–1731 (2023).

[18] Xu, L., Xia, C. & Chang, L. Dynamic modeling and vibration analysis of an RV reducer with defective needle roller bearings. *Eng. Fail. Anal.* **157**, 107884 (2024).

[19] Huang, J., Li, C. & Chen, B. Optimization design of RV reducer crankshaft bearing. *Appl. Sci.* **10**(18), 6520 (2020).

[20] Bartlomiej, A. et al. Intelligent diagnostics of radial internal clearance in ball bearings with machine learning methods. *Sensors* **23**(13), 5875 (2023). 37447725 10.3390/s23135875 PMC10346529

[21] Dalong, W., Ran, Li., Hao, L. & Jian, Ye. Wear analysis of slideway in emulsion pumps based on finite element method. *Sci. Rep.* **14**, 1930 (2024). 38253765 10.1038/s41598-024-51943-6 PMC10803306

[22] Rafal, R., Pawel, L., Krzysztof, K., Bogdan, K. & Jerzy, W. Chatter identification methods on the basis of time series measured during titanium superalloy milling. *Int. J. Mech. Sci.* **99**, 196–207 (2015).

